# Unbiased characterization of the larval zebrafish enteric nervous system at a single cell transcriptomic level

**DOI:** 10.1016/j.isci.2023.107070

**Published:** 2023-06-08

**Authors:** Laura E. Kuil, Naomi J.M. Kakiailatu, Jonathan D. Windster, Eric Bindels, Joke T.M. Zink, Gaby van der Zee, Robert M.W. Hofstra, Iain T. Shepherd, Veerle Melotte, Maria M. Alves

**Affiliations:** 1 Department of Clinical Genetics, Erasmus University Medical Center, Sophia Children’s Hospital, Rotterdam, the Netherlands; 2 Department of Pediatric Surgery, Erasmus University Medical Center, Sophia Children’s Hospital, Rotterdam, the Netherlands; 3 Department of Hematology, Erasmus MC, Rotterdam, the Netherlands; 4 Department of Biology, Emory University, Atlanta, GA, USA; 5 Department of Pathology, GROW-School for Oncology and Developmental Biology, Maastricht University Medical Center, Maastricht, the Netherlands

**Keywords:** Neuroscience, Developmental neuroscience, Transcriptomics

## Abstract

The enteric nervous system (ENS) regulates many gastrointestinal functions including peristalsis, immune regulation and uptake of nutrients. Defects in the ENS can lead to severe enteric neuropathies such as Hirschsprung disease (HSCR). Zebrafish have proven to be fruitful in the identification of genes involved in ENS development and HSCR pathogenesis. However, composition and specification of enteric neurons and glial subtypes at larval stages, remains mainly unexplored. Here, we performed single cell RNA sequencing of zebrafish ENS at 5 days post-fertilization. We identified vagal neural crest progenitors, Schwann cell precursors, and four clusters of differentiated neurons. In addition, a previously unrecognized *elavl3+/phox2bb-*population of neurons and *cx43+*/*phox2bb-*enteric glia was found. Pseudotime analysis supported binary neurogenic branching of ENS differentiation, driven by a notch-responsive state. Taken together, we provide new insights on ENS development and specification, proving that the zebrafish is a valuable model for the study of congenital enteric neuropathies.

## Highlights

• Single cell RNA sequencing of 5dpf zebrafish intestines uncovered 11 ENS subtypes
• Unbiased sequencing, reveals previously undescribed elavl3+/phox2bb-neurons
• Zebrafish larvae contain enteric glia expressing *her4, cx43, s100b*
• Zebrafish ENS differentiation branches into sensory IPANs and inhibitory motor neurons

## Graphical abstract

**Figure:**
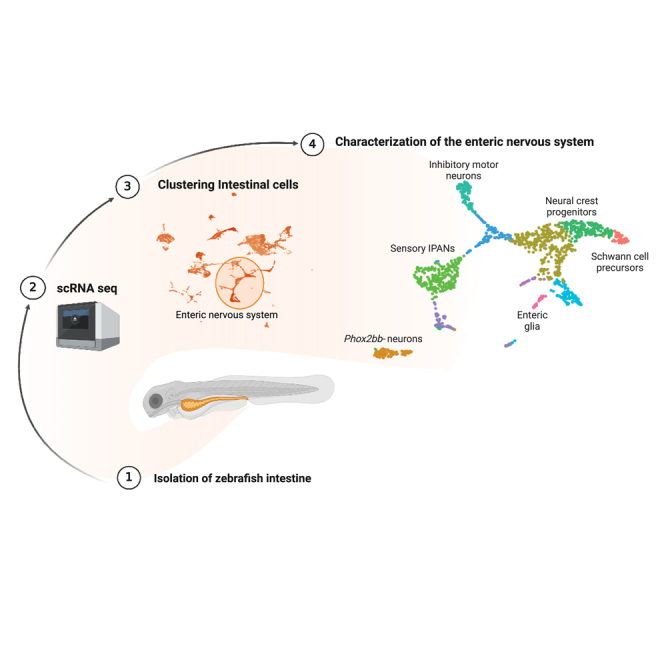

## Introduction

The enteric nervous system (ENS) consists of neurons and glia that are tightly interconnected, together and with cells in their microenvironment. The function of the ENS extends far beyond regulating peristalsis, as it is also involved in secretion, immune regulation and nutrient absorption via connections with other cell types in the intestine.^1^ It is well known that dysregulation of ENS development leads to life-threatening congenital enteric neuropathies, of which Hirschsprung disease (HSCR) is the most common disorder, affecting approximately 1 in 5,000 live births.^1^^,^^2^ ENS development occurs early during embryogenesis with vagal and sacral neural crest contributions. Recently, it has been found in mice that at postnatal stages, the ENS is supplemented by enteric neurons derived from Schwann cell precursors (SCPs).^3^ This suggests that there is a dual origin of ENS cells, namely those derived from embryonic (vagal) neural crest during early gut colonization, and those derived postnatally from SCPs.^3^

One of the vertebrate animal models that is regularly used to study ENS development, is the zebrafish.^4^ Zebrafish are highly suitable for genetic manipulation, develop rapidly, ex-utero and are transparent, which makes them extremely valuable for screening novel disease genes and tracing developmental processes.^5^^,^^6^ However, the precise composition and specification of different neuronal and glial subtypes in the zebrafish ENS, remains unclear. This holds true particularly at larval stages, in which key processes take place to ensure proper gut colonization with enteric neurons and glia. To date, a few immunohistochemistry studies investigating enteric neuronal identities in larval zebrafish, have reported the presence of vasoactive intestinal peptide (VIP), pituitary adenylate cyclase-activating peptide (PACAP), neuronal nitric oxide synthase (nNOS), serotonin (5-hydroxytryptamine; 5HT), calretinin (CR) and calbindin (CB), from 3 days post-fertilization (dpf) onwards.^7^^,^^8^ Recently, it has also been described that the adult zebrafish intestine contains enteric glia, similar to that observed in mammals.^9^ The study showed the presence of enteric glia presenting with neurogenic properties in the adult intestine, which could be detected by the notch reporter line *her4.3:*GFP.^9^ However, the presence of enteric glia in larval zebrafish, has still been poorly studied. Three papers reported contradicting findings regarding expression of canonical glial genes such as *gfap*, the traditional marker for enteric glia in human and mouse. Baker et al. showed Gfap+ enteric glia in the outer layer of the intestine of 7 and 18 dpf fish, encapsulated by a layer of enteric neurons.^10^ Transmission electron microscopy showed the presence of granular vesicles and filiform processes wrapping the muscularis and caveolae, which are typical characteristic of glia.^10^ McCallum et al. also showed Gfap+ staining in the larval intestine, but suggested that the immunostaining was aspecific, because it remained in the intestine of the *ret* mutant HSCR model, which lacks an ENS.^9^ Moreover, they showed that other typical enteric glial genes were not expressed in the zebrafish intestine, including *bfabp (fabp7a), sox10* and *s100b*.^9^ Such findings were supported by El-Nachef and Bronner, who reported the absence of enteric glia expressing *sox10, gfap, plp1a* and *s100b* in larval stages.^11^

To gain new insights into the exact ENS composition of larval zebrafish, studies at the single cell transcriptome level are warranted. Previous zebrafish studies described, did not capture enough neuronal cells for sub-analysis, or were done at very young embryonic and larval stages, showing limited neuronal specification.^12^^,^^13^^,^^14^ Here, we report single cell RNA sequencing (scRNA-seq) of 5 dpf zebrafish intestines. Importantly, we used an unbiased approach, dissecting whole intestines and sequencing all live cells without enrichment for specific ENS markers, such as *sox10* and/or *phox2bb*. Such an approach allowed detection of previously unrecognized neuronal and glial populations in the larval intestine at 5dpf, expanding our understanding of the ENS composition and specification in zebrafish.

## Results

### Vagal derived ENS cells are complemented by Schwann cell precursors (SCPs)

**Figure 1:**
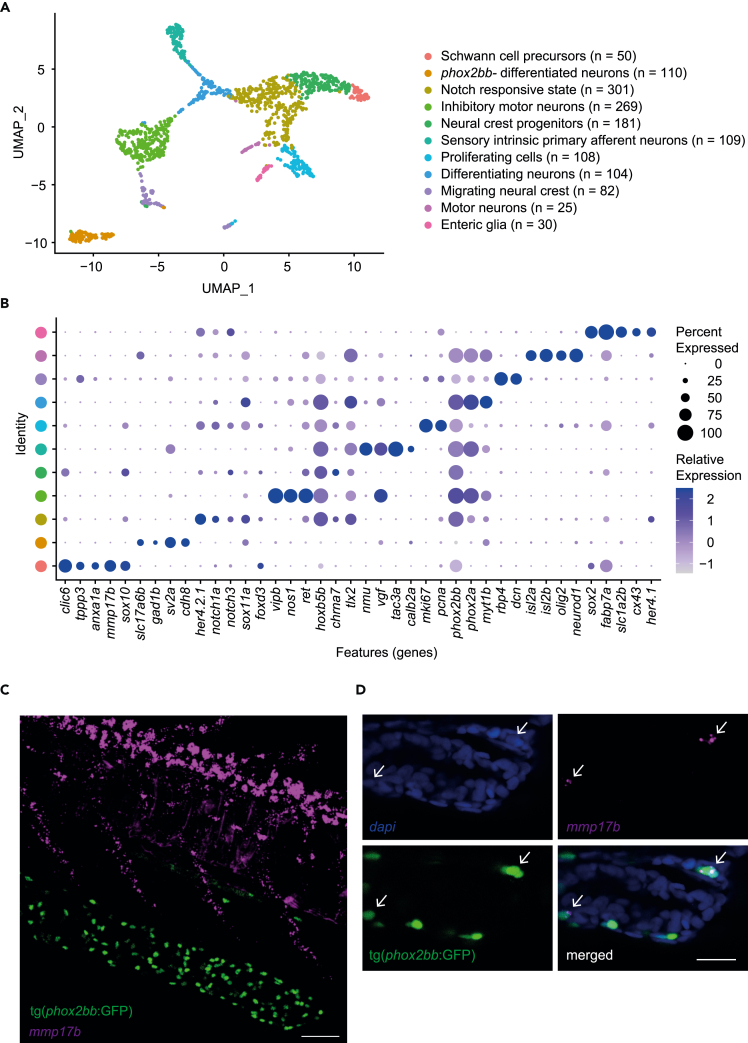
Single cell transcriptomics of 5 dpf zebrafish ENS (A) UMAP of 1369 ENS cells, containing eleven different clusters. (B) Dot plot showing expression of genes highly differentially expressed between clusters. (C) Maximum projection from FISH recordings of 5 dpf tg(*phox2bb*:GFP) larvae stained for *mmp17b* (magenta). Scale bar represents 20 μm. (D) Single plane detailed images of FISH of 5 dpf tg(*phox2bb*:GFP) larvae showing co-localization of dapi (blue), *mmp17b* (magenta) and phox2bb (green) in the intestine. Arrows highlight cells of interest showing co-localization. Scale bar represents 20 μm.

To enable capturing of the ENS from 5-day-old tg(*phox2bb*:GFP) larvae,^15^ 244 intestines were isolated (Figure S1A), dissociated and pooled to perform 10x scRNA-seq. The total intestinal dataset contained only one cluster of contaminant cells from the liver (Data S2). Based on expression of canonical markers and markers obtained in previous literature,^16^^,^^17^^,^^18^ we selected clusters that most likely contained neural crest progenitors, enteric neurons and glia (e.g. expression of *phox2bb, elval3/4, vipb, sox10, slc1a2b*) (Figures S1B–S1C).^14^^,^^19^ We further checked whether this subset exclusively contained ENS cells by clustering and analyzing differentially expressed markers. We identified some cycling immune cells (*lcp1*, *mki67*) and connective tissue cells (*col6a2*, *mki67*), which were manually excluded (Figures S1D–S1E).^20^^,^^21^ Analysis of the remaining cells (n = 1369 cells; 15% of total cells), led to eleven distinct clusters containing ENS cells (Figures 1A and 1B; Data S2). Two of these clusters were characterized by shared expression of typical neural crest/progenitor cell markers such as, *sox2/5/6/10, erbb3b and lama4* and were therefore, classified as progenitor cells (Figures 1B and, S2A).^22^^,^^23^^,^^24^ However, although one cluster selectively expressed genes typical for oligodendrocyte precursor cells (OPCs) or SCPs, including *clic6, tppp3, fabp7a, foxd3, col18a1a, lamb1b*, and *anxa1a*^16^^,^^25^^,^^26^^,^^27^^,^^28^^,^^29^ (n = 50 cells; Figures 1B and, S2B), the other cluster showed specific expression of well-known (vagal) neural crest genes, such as *ret, hoxb5b,* and *tlx2*^30^^,^^31^^,^^32^^,^^33^ (n = 181 cells; Figure 1B). *Mmp17b,* which has been described in migrating trunk neural crest and in Schwann cells on injury, was specifically identified in the SCP cluster (Figure S2B).^26^^,^^34^^,^^35^ We then performed fluorescent whole mount *in-situ* hybridization (FISH) using a probe targeting *mmp17b*, to localize these cells in 5 dpf zebrafish and determine if they are specifically present in the gut. As expected, *mmp17b* positive cells were present in the spinal cord and in the axonal motor neuron branches, corresponding to the known localization of SCPs (Figure 1C).^26^^,^^36^^,^^37^ In the intestine, *mmp17b* signal was also observed, occasionally co-localizing with the *tg(phox2bb:*GFP) signal inside the intestine (Figure 1D and, Video S1). This signal was sparse, which is in line with our scRNA-seq data, where the majority of *mmp17b* positive cells (32 out of 52 cells) showed only 1 or 2 RNA counts/cell. To support the SCP identity of these cells, we performed FISH using a *mmp17b* probe and a *sox10* probe as an additional SCP marker. Co-localization of *sox10*, *mmp17b* and tg(*phox2bb*:GFP) signal was observed in the intestine of 5 dpf tg(*phox2bb*:GFP) zebrafish (Figure S2C).Therefore, our results confirm the presence of SCPs in the zebrafish intestine,^11^ and support the rare nature of these cells at 5dpf.

Video S1. Video of a z-stack showing tg(phox2bb:GFP) in green and FISH mmp17b in magenta, showing co-localization of the two markers, related to Figure 1

### The zebrafish intestine contains four types of differentiated neurons at larval stage

**Figure 2:**
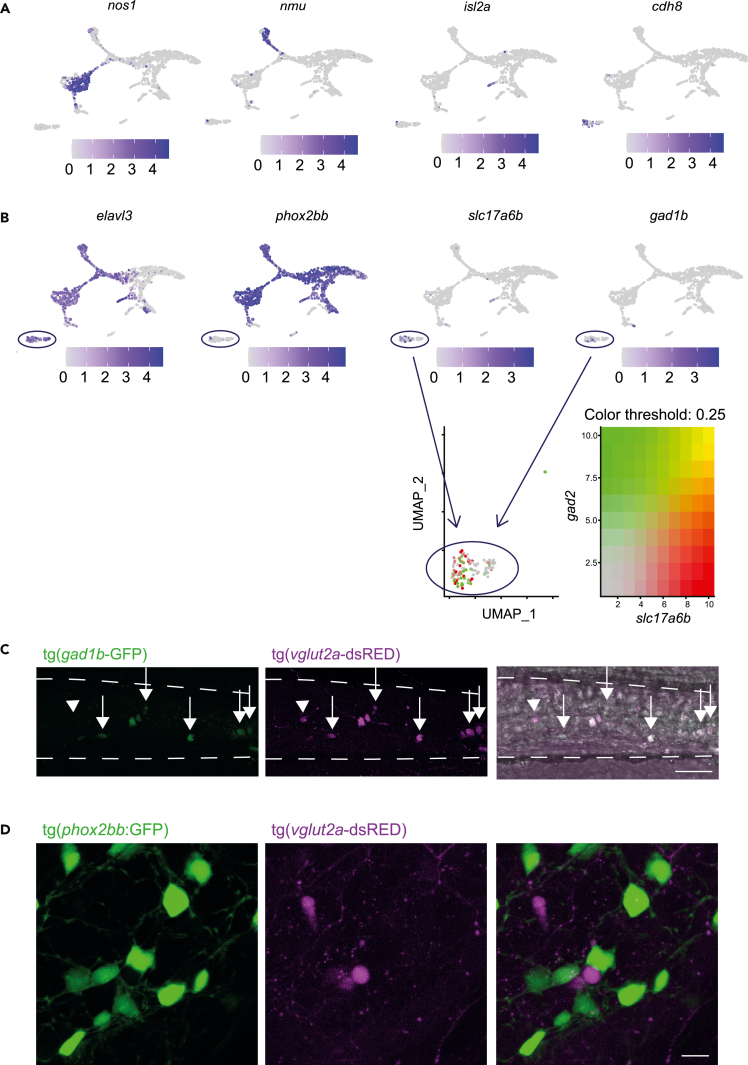
The 5 dpf intestine contains four clusters of differentiated neurons including a cluster of elavl3+; phox2bb-enteric neurons showing excitatory and inhibitory gene expression (A) Featureplots of genes defining four clusters of differentiated neurons. (B) Featureplots highlighting the presence of a cluster of cells expressing *elavl3*, *slc17a6b* and *gad1b*, but lacking expression of *phox2bb* (*phox2bb*-differentiated neurons, depicted by the circle). (C) Maximum projections of live imaging recordings from 7 dpf tg(*gad*1b:GFP); tg(*vglut2a*-dsRed) larval intestine shows overlap between the two reporters (arrows) with one gad1b+ cell that is vlgut2- (arrowhead). Scale bar represents 40 μm. (D) Maximum projections of live imaging recordings from 7 dpf tg(*phox2bb*:GFP); tg(*vglut2a*-dsRed) larval intestine shows no overlap between the two reporters. Scale bar represents 20 μm.

**Figure 3:**
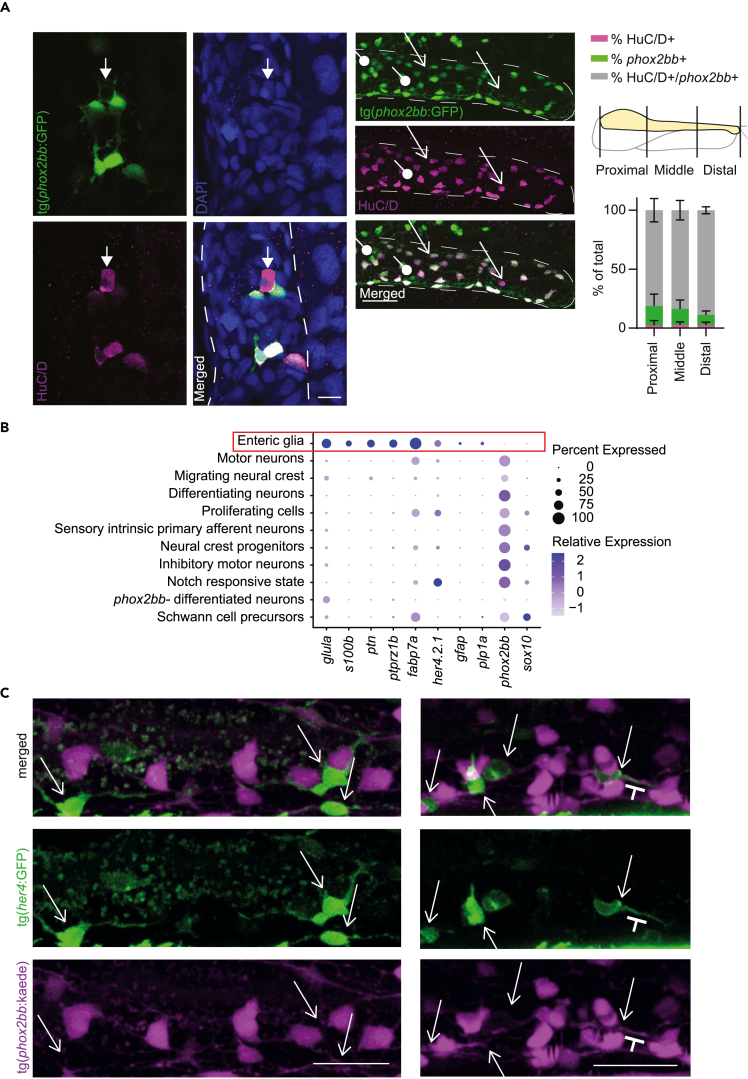
One small cluster expresses genes typical for enteric glia in mammalians (A) Left: Single plane recording showing a HuC/D+phox2bb-neuron. Scale bar represents 10 μm. Right: Maximum projections of HuC/D antibody staining shows that most HuC/D+ cells in the intestine express phox2bb, but also show phox2bb+/HuC/D-cells (progenitors) depicted by the arrows with a circle end, and phox2bb-;HuC/D+ cells (differentiated neurons) depicted by arrows. Scale bar represents 40 μm. Quantification of the relative amount of double and single positive cells, relative to the total number of enteric neurons (HuC/D only, phox2bb only and double-positives combined)(n=9; 5 dpf, error bars show standard deviation). (B) Dotplot showing selective expression of some known enteric glia and radial glia markers and lack of expression of *phox2bb* and *sox10* in the cluster of enteric glia depicted by the red box. (C) Maximum projections of live-imaging recordings of 5 dpf photoconverted tg(8.3*phox2bb*:kaede);tg(*her4*:GFP) intestines shows phox2bb-;her4+ cell depicted by the arrows, that are in close proximity to, or seem to interact with phox2bb+ neurons (magenta; close interaction between extensions is depicted by the T mark). Scale bar represents 20 μm.

Based on our clustering settings (Seurat pipeline using 6 dimensions and 0.4 resolution), four types of ‘differentiated neurons’ were identified. The largest cluster (n = 269 cells) consisted of inhibitory motor neurons, expressing *vip* and *nos1* (Figures 1B and 2A).^29^^,^^38^ This cluster also included cells expressing *slc6a4b*, *tph1b* and *ddc,* which are genes involved in serotonin transport and production (Figure S3A).^39^^,^^40^^,^^41^ Sensory intrinsic primary afferent neurons (IPANs) were identified by expression of *nmu*, *vgf, tac3a* and *calb2a* (n = 109 cells; Figures 1B and, 2A).^29^^,^^42^ A third cluster expressing *isl2a/b, olig2* and *neurod1*, most likely represents motor neurons (n = 25 cells; Figures 1B and 2A).^43^^,^^44^^,^^45^ The fourth cluster seemed to contain a mix of different neuronal subtypes such as, glutamatergic, GABAergic and IPANs (n = 110 cells), based on their selective expression of *vglut2a (slc17a6b),* *gad1b, sv2a* and *cdh8* (Figures 1B and 2A–2B).^29^^,^^46^^,^^47^ Of interest, the latter cluster did not express *phox2bb* (Figure 2B). To confirm the presence of *phox2bb*-neurons in the zebrafish intestine, we used two reporter lines, the tg(*vglut2*:loxp-dsRed-loxP-GFP) and the tg(*gad1b*:GFP). Expression of both *VGlut2* and *gad1b* is known to be specific to neuronal cells in the brain. Here, we show that these two reporters are also expressed in the intestine, although at low levels. In contrast to what is observed in the brain and in our transcriptomic data, *vglut2* and *gad1b* expression co-localized in the majority of cells (Figure 2C). Subsequently, we crossed the tg(*phox2bb*:GFP) reporter with the tg(*vglut2*:loxp-dsRed-loxP-GFP) reporter and found no co-localization between *phox2bb* and *vglut2 in vivo,* confirming the presence of *phox2bb-*/*vglut2a+* cells in the zebrafish intestine (Figure 2D). Our transcriptomic data also showed that these cells express *elavl3* (encoding HuC; Figure 2B), which led us to perform a HuC/D staining on tg(*phox2bb*:GFP) fish. A limited number of HuC/D+;*phox2bb*-cells (between 0 and 25) were observed in the zebrafish intestine, comprising on average 2.5% of the total number of enteric neurons (Figure 3A; Video S2). Distribution of HuC/D+;*phox2bb*-cells seems equal along the anterior to posterior axis, indicating that these cells are evenly distributed along the total length of the intestine and therefore, do not seem to be region specific (Figure 3A). To confirm their localization, we performed intestinal isolation after immunohistochemistry showing that HuC/D+;*phox2bb*-cells are indeed located in the intestine (Figure S3B).

Video S2. Video of a z-stack showing tg(phox2bb:GFP) in green and HuC/D immunohistochemistry in magenta, showing a HuC/D + phox2bb-cell adjacent to phox2bb+ cells in the intestine, related to Figure 3

### Larval zebrafish intestine contains enteric glia

**Figure 4:**
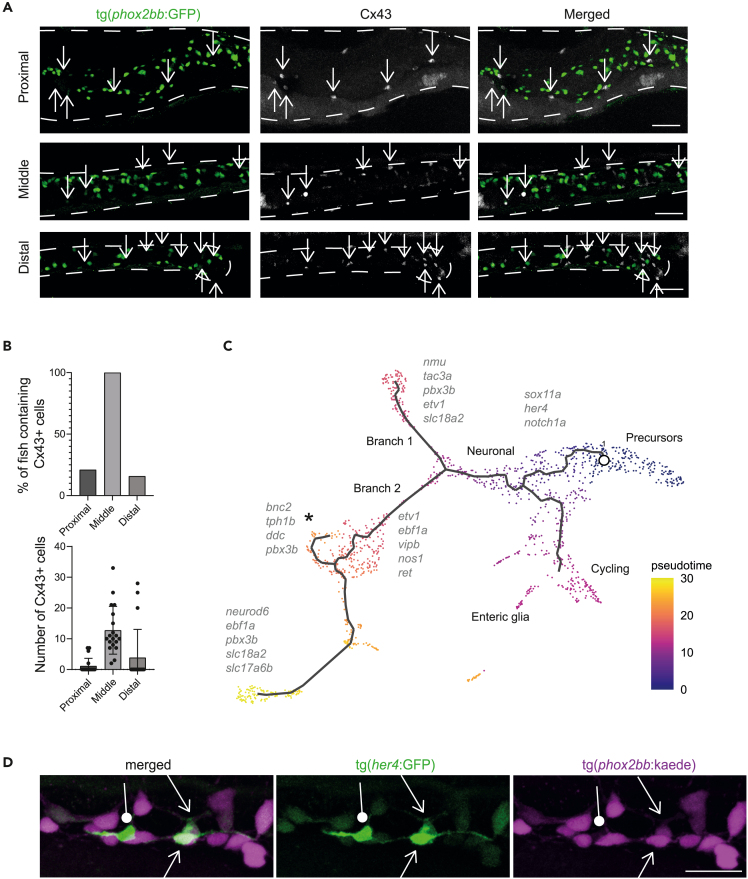
Pseudotime analysis shows differentiation trajectories from right to left of the UMAP (A) Immunohistochemistry staining of Cx43 in the tg(*phox2bb*:GFP) reporter line shows non overlapping expression in the intestine. Representative maximum projections from 4 dpf larvae. Squares depict the area of the detailed zoom image. Arrows depict Cx43+phox2bb-enteric glia. Scale bar represents 50 μm. (B) Upper graph shows the percentage of larvae that contained Cx43 cells in their proximal, middle and distal intestine. The lower graph shows the number of Cx43 cells per larvae in the proximal, middle and distal intestine (n = 19, error bars show standard deviation). (C) Pseudotime color-coded featureplot showing a bifurcation toward neuronal differentiation (sensory IPAN: branch1 and inhibitory motor neurons: branch2 containing a secondary branch toward serotonergic neurons marked with an asterix). (D) Maximum projections of live-imaging 5 dpf tg(8.3*phox2bb*:kaede); tg(*her4*:GFP) intestines shows phox2bb+; her4+ cells (arrows), representing cells undergoing differentiation from progenitor state toward neuronal or glial fate. The arrows with a circle end points to enteric glia. Scale bar represents 20 μm.

Although recent compelling evidence suggest the presence of enteric glia in the adult zebrafish intestine,^9^ the presence and characterization of these cells in larval zebrafish has not been extensively described. This is mainly because of the absence in the fish of expression markers identified in mice and humans, typical for this cell type. In line with previous reports, our data confirmed the absence of *gfap+* cells in the larval intestine of the tg(*gfap*:GFP) reporter line (Figure S3C). However, due to our unbiased approach of sequencing total intestines, we were able to observe a cluster of cells lacking expression of *phox2bb* and *sox10,* but expressing Hairy/E(spl)-related 4 (*her4*) genes (n = 30 cells; Figure S3D). Of interest, this cluster showed highly specific expression of genes typically found in radial glia in the zebrafish brain, such as *glula, slc1a2b* and *ptn*,^16^ and of genes expressed in mammalian enteric glia, such as *cx43*, *s100b, sox2, ptprz1b,* and *fabp7a* (Figures 1B and, 3B).^48^^,^^49^^,^^50^^,^^51^^,^^52^ By photoconversion of tg(*phox2bb*:kaede) in larvae also containing the tg(*her4*:GFP) construct, we showed that *her4+*;*phox2bb*-cells are indeed present in the intestine and are located in close proximity, or in some cases in direct contact to *phox2bb*+ cells (Figure 3C). To validate the enteric glial identity of these cells, we performed immunohistochemistry on tg(*phox2bb:*GFP) larvae using an antibody against connexin 43 (Cx43), a known enteric glia marker in mice that we found expressed in the *Cx43+*/*phox2bb-*cluster. Based on our results, Cx43+/*phox2bb*-cells were detected at 4 dpf, but not at 3 dpf, suggesting that enteric glia start to express Cx43 between 3 and 4 dpf in zebrafish (Figure 4A). Cx43+ cells were most often observed in the middle intestinal segment, with an average of 12 cells per fish (Figure 4B). Although the location of Cx43+ cells is similar to *phox2bb*+ cells, as they were often observed in the same focal plane in close proximity to each other, these cells were always negative for the *phox2bb* reporter. Furthermore, these cells were negative for *sox10*, which is in line with earlier findings (FigureS4).^9^

### Progenitor cells become notch-responsive before differentiation toward neuronal and glial fate

Analysis using monocle3^53^ showed a Pseudotime trajectory from progenitors to differentiated neuronal clusters (Figure 4C). During differentiation, a bifurcation into two types of early differentiated neurons occurs, branch 1: sensory IPANs versus branch 2: inhibitory motor neurons (Figure 4C). This latter branch also contains a secondary branch toward serotonergic neurons (see asterix in Figure 4C). Morarach et al. reported a similar bifurcation in the murine ENS differentiation trajectory, with branch A forming *Vip/Nos* positive neurons.^29^ Expression of “branch A marker genes” *etv1* and *ebf1a*, was found in our dataset, in the *vip+/nos1+* inhibitor motor neuron branch (Figure 4C). However, expression of most “branch B marker genes” was either absent or not specific to one branch in our dataset. Only *bnc2* was expressed in the serotonergic cells in the inhibitory motor neuron cluster (Figure 4C).^29^ Comparing our data to the dataset from Howard et al. showed that the early differentiation observed in zebrafish from 68 to 70 h post-fertilization (hpf), is prominently seen in our dataset as well.^14^ For example, co-expression of *slc18a2* and *pbx3b* was observed in differentiated neurons, including IPANs (Figure 4C).

Our data also showed that neuronal differentiation seems to start in the notch-responsive cell cluster, which specifically expresses notch receptors (*notch1a*, *notch3*)*,* the upstream regulator of notch (*lfng*)*,* and various downstream transcription factors (e.g. *her4.2.1, her4.2, her4.3*) (Figures 1B and S3D).^9^ In line with these results, co-expression of the known cell fate mediator gene *sox11a*, and notch downstream transcription factors was observed (Figure 1B).^16^ The tg(*her4*:GFP) line is a reporter line used to visualize expression of notch downstream transcription factors and thus serves as a reporter for notch signaling.^54^ Live-imaging of 5 dpf tg(*her4*:GFP)/tg(8.3*phox2bb*:kaede) fish showed that a subset of *phox2bb*+ cells co-express *her4*+ (15% of cells in the posterior intestine), confirming the presence of *phox2bb*+*/her4*+ notch responsive cells *in vivo* (Figure 4D), and corroborating previous work.^9^ Thus, progenitor cells seem to become notch responsive on initiation of differentiation toward neurons (branch 1 and 2). In addition, there appears to be a third trajectory emanating progenitors toward clusters containing proliferative cells, motor neurons and enteric glia, suggesting a separate differentiation route toward a cycling/enteric glial fate (Figure 4C).

## Discussion

Here, we present for the first time a single cell atlas of the ENS of 5 dpf zebrafish. Our results show the presence of two clusters of progenitor cells, traditional vagal neural crest cells and SCPs. Based on our dataset, we were able to identify *mmp17b* as a specific marker for SCPs, and were able to confirm the presence of these cells in the gut, as well as their rare nature *in vivo*. Our data also showed the presence of four clusters of enteric neurons and a cluster of enteric glia, at this developmental stage. Inhibitory motor neurons were confirmed to be the major neuronal population present in the zebrafish ENS,^7^^,^^8^ but we were also able to identify other differentiated neuronal subtypes. We observed that early enteric neuronal differentiation occurs as an initial bifurcation toward two major branches. Sensory IPANs seem to develop via branch 1, whereas *vip+/nos1+* inhibitory motor neurons specify via branch 2. The latter contains a secondary branch toward serotonergic enteric neurons, which might resemble branch B identified in mice based on *bnc2* expression.^29^ Therefore, differentiation of enteric inhibitory motor neurons and serotonergic neurons seems to be conserved between, at least, mice and zebrafish.^29^ Of interest, because of our unbiased sequencing approach in which the whole intestine was analyzed, we were able to identify one cluster of *elavl3+*/*phox2bb*-differentiated neurons, expressing genes specific for glutamatergic neurons, GABAergic neurons, and others involved in serotonergic signaling. Although we could not exclude the possibility of contamination with non-intestinal cells in our transcriptomics data, based on our immunohistochemistry and live-imaging data, *elavl3+*/*phox2bb*-neurons are located inside the gut, in close proximity or sometimes even directly adjected to *phox2bb+* enteric neurons. To our knowledge, such a population has never been defined before, as all enteric neurons were assumed to express *phox2bb*. Future lineage tracing experiments should be performed to confirm the neural crest-origin of these cells, as well as scRNA-seq experiments at older ages, to provide insights into which neuronal sub-types these *phox2bb*-cells contribute.

Finally, our dataset showed the presence of a cluster of enteric glial cells. In line with previous studies, we found that the relative contribution of enteric glia to the ENS seems to be less abundant in zebrafish, compared to that in human and mice.^24^^,^^55^ We now show that enteric glia can be detected already at zebrafish larval stages and express some less well-known (enteric) glial genes (*cx43, fabp7a, s100b*) while lacking expression of *sox10, phox2bb* and *plp1a*. These cells do express the *her4* reporter line, described before to label zebrafish enteric glia.^9^ Importantly, we show that only the *phox2bb-*/*her4+* cells, but not the *phox2bb+/her4+* cells, express enteric glial markers at larval stages and thus can be assumed to be differentiated enteric glia. Of interest, the *phox2bb*+/*her4+* cells lack expression of glia markers, but express notch receptors, notch regulators and notch downstream transcription factors and thus, are referred to as ‘notch-responsive state’ in our data. However, because they lack expression of glia markers their definitive identity has to be further defined. Pseudotime analysis shows that *phox2bb*+/*her4+* cells are initiating differentiation toward a neuronal or glial fate. This is in line with the McCallum study showing that enteric neural crest cells start to express the *her4* reporter after migration, and lose this expression on differentiation.^9^ Here, we extended their findings by showing differentiation at a single cell transcriptional level, from progenitors via a Notch responsive state, toward early specification of enteric neuronal fate. This suggests that Notch signaling plays a central role in the transition from progenitor to neuronal state or glial differentiation. In line with this, disruption of Notch signaling in mice (*Pofut1* knockout), was shown to result in the absence of an ENS, confirming that this signaling pathway is crucial to maintain the neural crest progenitor pool.^56^ The Notch pathway has also been recognized in the maintenance of neuronal stem cells in the brain, but signaling dynamics in neuronal differentiation have yet to be elucidated (Reviewed by^57^).

Taken together, our results show that the zebrafish ENS contains vagal neural crest and SCPs that follow specification toward either a neuronal fate, or via a cycling state toward an enteric glial fate. It also shows that using an unbiased approach in which cells are not selected for a specific reporter construct, can be instrumental in finding new cell clusters. In summary, our data adds to the understanding of healthy ENS development and offers an essential framework for intra-study, cross-species, and disease state comparisons.

### Limitations of the study

One limitation of this study is the relatively low number of ENS cells captured by scRNA-seq. Therefore, we may not fully capture the heterogeneity and complexity of the larval zebrafish ENS. Increasing the number of cells in future studies would enhance the comprehensiveness and robustness of our findings. Furthermore, although we identified a previously undescribed population of *elavl3+/phox2bb*-neurons, its functional characterization was not explored. Future research is therefore necessary to explore the role of this new population in intestinal function.

## STAR★Methods

### Key resources table

**Table:** 

REAGENT or RESOURCE	SOURCE	IDENTIFIER
**Antibodies**		

HuC/HuD Neuronal Protein Mouse Monoclonal Antibody	Molecular Probes/Thermofisher	Cat#A-21271; RRID:AB_221448
Connexin-43 (E7N2R) XP Rabbit mAb	Cell Signaling Techn	Cat#83649

**Chemicals, peptides, and recombinant proteins**		

Anti Mouse Cy3	Jackson	Cat#715-165-150; RRID:AB_2340813
Monovalent AffiniPure Fab Fragment Cy3	Jackson	Cat#711-167-003; RRID:AB_2340606
Donkey anti-rabbit Alexa Fluor 488	Thermofisher	Cat#A-21206; RRID:AB_2535792
Goat- anti-mouse Alexa Fluor 568	Thermofisher	Cat#A-11004; RRID:AB_2534072
DAPI	Sigma-Aldrich	Cat#D-9542
Ultrapure Low melting	ThermoFisher	Cat#16520050
MS-222, Tricaine	Supelco	#A5040-250G
Papain	Sigma	Cat#P4762
HBSS with CaCl2 and MgCl2	Gibco	Cat#14025050
L-Cystein	Sigma	Cat#C7352
Tween 20	Sigma	Cat#P7949-500
Triton X-100	Sigma	Cat#T8787
BSA	Sigma-Aldrich	Cat#A3294
Horse serum	Invitrogen	Cat#16060-122
Glycerol	Riedel-de Haën	Cat#49770-1L-D
Trypan blue	Sigma	Cat#T8154

**Critical commercial assays**		

RNAscope Multiplex Fluorescence Reagent Kit v2 Assay	ACDbio	Cat#323100
10X Chromium Single Cell 3′ Kit	10X Genomics	Cat# 120237
Mmp17b probe	ACDbio	Custom
Opal 570 dye	Akoya	SKU FP1488001KT

**Deposited data**		

Raw single-cell RNA-seq data	This study	GEO: GSE225510
Processed single-cell RNA-seq data	This study	GEO: GSE225510

**Experimental models: Organisms/strains**		

Zebrafish tg(*phox2bb*:GFP)	Nechiporuk et al. (2007)	ZDB-TGCONSTRCT-070522-1
Zebrafish tg(*8.3phox2bb:kaede*)	Harrison et al. (2014)	ZDB-TGCONSTRCT-150305-1
Zebrafish tg(*her4*:GFP)	Sang-Yeob et al.(2007)	ZDB-TGCONSTRCT-070612-3
Zebrafish tg(*gfap*:GFP)	Raymond et al. (2006)	ZDB-TGCONSTRCT-070117-154
Zebrafish tg(*vglut2*:loxp-dsRed-loxP-GFP)	Miyasaka et al. (2009)	ZDB-TGCONSTRCT-070117-143
Zebrafish tg(*gad1b*:GFP)	Satou et al. (2013)	ZDB-TGCONSTRCT-131127-6

**Software and algorithms**		

Cell Ranger v3.0.2	10X Genomics	N/A
Fiji	ImageJ	National Center for Microscopy and Imaging Research: ImageJ Mosaic Plug-ins, RRID:SCR_001935
Leica	LASX	Leica Application Suite X, RRID:SCR_013673
Adobe illustrator	Adobe Inc.	N/A
Seurat v3	Stuart et al. (2019)	https://satijalab.org/seurat/
Monocle 3	Trapnell et al. (2014)	https://cole-trapnell-lab.github.io/monocle3/
R version 4.0.3	R Core Team (2022)	https://www.R-project.org/
RStudio 1.2.5033	RStudio Team (2020)	http://www.rstudio.com/
Prism 5	Graphpad	GraphPad Prism, RRID:SCR_002798

### Resource availability

#### Lead contact

Further information and requests for reagents may be directed to, and will be fulfilled by Maria M. Alves (m.alves@erasmusmc.nl).

#### Materials availability

This study did not generate new, unique reagents.

### Experimental model and study participant details

#### Animal husbandry

The following zebrafish lines were used: transgenic tg(*phox2bb*:GFP),^15^ tg(*8.3phox2bb:kaede)*,^58^ tg(*her4*:GFP),^54^ tg(gfap:GFP),^59^ tg(*vglut2*:loxp-dsRed-loxP-GFP),^60^ and tg(*gad1b*:GFP).^61^ Zebrafish were kept on a 14/10h light/dark cycle. Embryos and larvae were kept in an incubator at 28.5°C in HEPES-buffered E3 medium. For imaging experiments, fish were treated from 24 hpf onwards, with 0.2 mM 1-phenyl 2-thiourea (PTU), to inhibit pigmentation. Animal experiments were approved by the Animal Experimentation Committee of the Erasmus MC, Rotterdam.

### Method details

#### Isolation of zebrafish intestines

Intestines of 5 days post-fertilization (dpf) larvae were isolated as followed: a row of 6–10 larvae anesthetized with 0,0016% Tricaine, were placed on an 1.8% agarose plate. Intestines were isolated using insect pins under a dissection microscope (Olympus SZX16). Non-intestinal tissue was manually removed if needed (Figure S1A). The intestine was collected with a tweezer and placed in an Eppendorf tube containing phosphate buffered saline (PBS) with 10% fetal calf serum (FCS) on ice. In total 244 intestines were isolated and pooled together. The total dissection time was kept at 3 hours.

#### Pre-processing of zebrafish cell suspension for scRNA sequencing

Immediately after dissections, the intestines were centrifuged at 17000g for 30 seconds and the PBS/FCS10% was replaced for 2.17 mg/mL papain dissolved in HBSS, with CaCl_2_ and MgCl_2_ to dissociate the cells. Papain was activated using 2.5μl cysteine (1M) and dissociation was performed in a water bath at 37°C, for 10 minutes, by pipetting up and down after 5 minutes to stimulate digestion. Cells were then transferred into a FACS tube using a 35 μm cell strainer and centrifuged at 700g for 5 minutes at 4°C, The supernatant was removed and pellets were resuspended in PBS containing 10% FCS. DAPI was added to mark dead cells (1:1000). All sorts were performed using the FACSAria III sorter, into eppendorfs containing PBS with 5% FCS to sort for live, single cells. The cell suspension was counted with a hemocytometer, including a viability check with Trypan blue.

#### Single cell RNA sequencing (scRNA-seq)

Single cells were barcoded using a 10x genomics Chromium Controller, and sequenced using a Novaseq 6000 instrument (Illumina). cDNA was prepared using the manufacturer’s protocol (Chromium [version 2]; 10× Genomics). Data was mapped using cell ranger V3.0.2 (GRCz10). In total, 9,858 cells were sequenced with mean reads per cell of 21,106. For scRNA-seq analysis we used Seurat V3.^62^^,^^63^^,^^64^ The Seurat pipeline was used for filtering (nFeature_RNA > 100 & nFeature_RNA <4200 & percent.mito < 0,05), normalization and downstream analysis for clustering, where we used 50 dimensions with a resolution of 0,8 for the UMAP processing. This led to 49 clusters, which we annotated based on differential gene expression. Seven clusters expressing neuronal and/or enteric progenitor markers were identified (cluster 6, 10, 13, 19, 21, 23, 34) (Figures S1A–S1B). These seven clusters were selected for a subset analysis, using 30 dimensions with a resolution of 0,7 for the clustering and UMAP, resulting in 14 clusters. One cluster was excluded as it contained leukocytes (*lcp1*+, *phox2bb-, elavl3-, sox10-*; cluster 12). Proliferating cells negative for *phox2bb* and *elavl3*, but expressing *mki67* and *col6a2* and likely to be connective tissue were also manually excluded (Figures S1C–S1D). The final set was analyzed using six dimensions and a resolution of 0,4. This analysis provided us with eleven clusters, which were annotated based on the differential gene expression and literature search. If required, a more thorough analysis of the total set of differentially expressed genes per cluster, or differentially expressed genes between specific subclusters, was performed. For pseudotime analysis, monocle3 was used^53^ using the Seurat processed data as input retaining the same clustering. The SCP cluster was chosen as the root node and our initial clustering was maintained for the pseudotime analysis.

#### Immunohistochemistry

Whole mount immunohistochemistry using mouse anti-HuC/D (1:100, molecular probes A-21271) was performed, as previously described.^65^ Zebrafish were fixed overnight in 4% PFA at 4°C. They were dehydrated in 70% EtOH at 4°c overnight. Next, the samples were rinsed 3x with PBS, followed by 3 × 30 minutes rinsing with double-distilled water. They were incubated for 2 hours with a blocking solution (0,2%BSA/0,5%Triton X-100/1%DMSO/5%horse serum/PBS) to reduce non-specific binding. Subsequently, they were incubated overnight in anti-HuC/D diluted antibody in the blocking solution at 4°c. The next day, the primary antibody was removed, samples were rinsed 3 × 30 minutes with PBS containing 0,1% Triton X-100 and incubated overnight in blocking solution containing 1:250 anti-mouse cy3 at 4°c. Finally, samples were rinsed 3 × 30 minutes with PBS containing 0,1% Triton X-100, then incubated with DAPI for 1 minute and rinsed again for 3 × 5 minutes with PBS. Samples were stored in PBS at 4°c. For Figure S3B additional primary antibody rabbit anti-GFP (1:100, Thermofisher) and secondary antibody donkey anti-rabbit Alexa Fluor 488 (1:500, Thermofisher) were used and intestines were dissected from the larvae after staining.

The antibody staining using rabbit anti-Cx43 (1:200, Cell Signaling Technologies 83649) was performed as published before.^66^ Zebrafish were fixed overnight in 4% PFA at 4°c. Samples were rinsed with 1% SDS/PBS for 5 minutes while rocking at room temperature (RT). Subsequently, samples were rinsed in PBST (PBS + 0,1% Tween), incubated in PBS + 0,1% Tween + 0,1% Triton X-100 (PBSTX) for 1 hour and incubated in 10%BSA/PBSTX for 1 hour to reduce non-specific binding. Then, samples were incubated at 4°c for 48 hours in primary antibody Cx43 including monovalent AffiniPure Fab Fragments (111-167-003 Jackson; Cy™3 AffiniPure Fab Fragment Goat Anti-Rabbit IgG) to increase signal to noise ratio.The AffiniPure Fab Fragments was incubated with the primary anti-Cx43 for 10 minutes before use.

#### Single-molecule whole-mount fluorescent RNA *in situ* hybridization

Zebrafish were fixed in 4% paraformaldehyde in PBS, overnight. They were then dehydrated through a series of 25/50/75/100% MeOH in PBST for 5 minutes each, and stored for a minimum of 1 hour at −20°c. Next, samples were rehydrated through a series of 75/50/25/0% MeOH in PBST for 5 minutes each, and incubated in prot K for 15 minutes at 20°c. They were rinsed twice with PBST for 5 minutes and re-fixed in 4% PFA in PBS, for 20 minutes at RT. Subsequently, samples were rinsed again 5 × 5 minutes in PBST. After manual pre-treatment for permeabilization, the RNAscope Multiplex Fluorescence Reagent Kit v2 Assay (Advanced Cell Diagnostics, Bio-Techne), was used according to the manufacturers’ instructions. A custom made probe for dr-*mmp17b* C1 (NPR-0035110) and a dr-s*ox10* C2 probe (Bio-techne 444691-C2) were used (Advanced Cell Diagnostics, Bio-Techne). TSA Vivid Fluophore Kit 570 and TSA Vivid Fluophore Kit 620 (Tocris Bioscience) were used for channel development.

#### Fluorescent imaging

Imaging was performed as previously described, using the Leica SP5 intravital microscope with a 20x water-dipping lens.^67^ For the kaede photoconversion experiments (co-localization of *her4*:GFP and *phox2bb*:kaede), all *phox2bb+* cells in the total intestines were photoconverted using the 405 nanometer (nm) laser, as described earlier.^11^ After photoconversion, the green and red channels were recorded, using a sequential scan with the 488nm and 561nm lasers to confirm full photoconversion. For Figure S3B, images were taken using an Olympus spinning disk attached to an IX81 microscope. Images were captured using 3i Slidebook 6.0 software.

### Quantification and statistical analysis

The immunohistochemistry, smRNA ISH and live-imaging recordings were quantified using FIJI (ImageJ2, https://imagej.net/) by carefully assessing all focal planes to unsure co-localization, or lack thereof.

## Data Availability

• The raw and processed data used for this study are available in NCBIs Gene Expression Omnibus though GEO series accession number GSE225510 https://www.ncbi.nlm.nih.gov/geo/info/linking.html. • This paper does not report original code. • Any additional information required to reanalyze the data reported in this paper is available from the lead contact upon request.

## Supplemental information

Document S1. Figures S1–S4

Data S1. Table showing the top10 positive markers for all the clusters in the total intestinal dataset, related to Figure S1

Data S2. Table showing the top10 positive markers for all the ENS-subset clusters, related to Figure 1
