# Supplementary material for: Unbiased characterization of the larval zebrafish enteric nervous system at a single cell transcriptomic level
Source: iScience. 2023 Jun 8;26(7):107070. doi: 10.1016/j.isci.2023.107070 (PMC10329177; doi:10.1016/j.isci.2023.107070)
Supplement: Document S1. Figures S1–S4 [file mmc1.pdf]

iScience, Volume *26*

Supplemental information

Unbiased characterization of the larval zebrafish enteric nervous system at a single cell transcriptomic level

Laura E. Kuil, Naomi J.M. Kakiailatu, Jonathan D. Windster, Eric Bindels, Joke T.M. Zink, Gaby van der Zee, Robert M.W. Hofstra, Iain T. Shepherd, Veerle Melotte, and Maria M. Alves

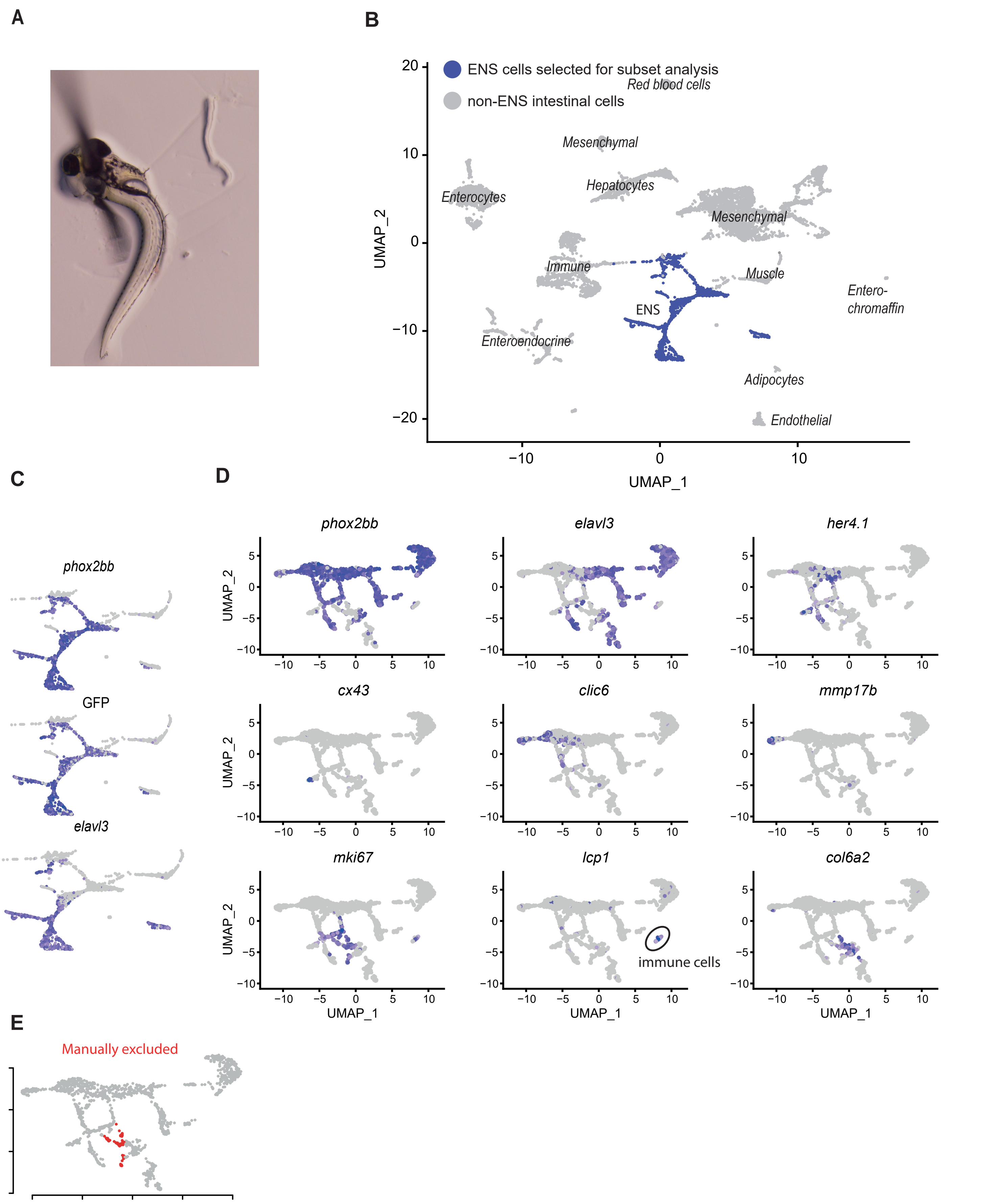

Figure S1. Isolation of zebrafish intestine and featureplots showing the clusters selected for subset analysis of the ENS, related to Figure 1

A) Brightfield image of intestinal isolation from a 5 dpf zebrafish larvae using insect pins. B)UMAP featureplots showing the cells that are selected for subset analysis of the ENS in purple and other non-ENS cells in grey. C) Featureplots showing expression of phox2bb, GFP, and *elavl3* in this part of the UMAP. D) Featureplots showing expression of various known ENS markers in the subset, but also expression of the immune marker *lcp1* and connective tissue marker col6a2, which colocalizes with the proliferative marker *mki67*. E) manual removal of the connective tissue cells marked in red colour. The *lcp1+* cells formed a separate cluster, thus did not require manual annotation of cells to be removed from the selection.

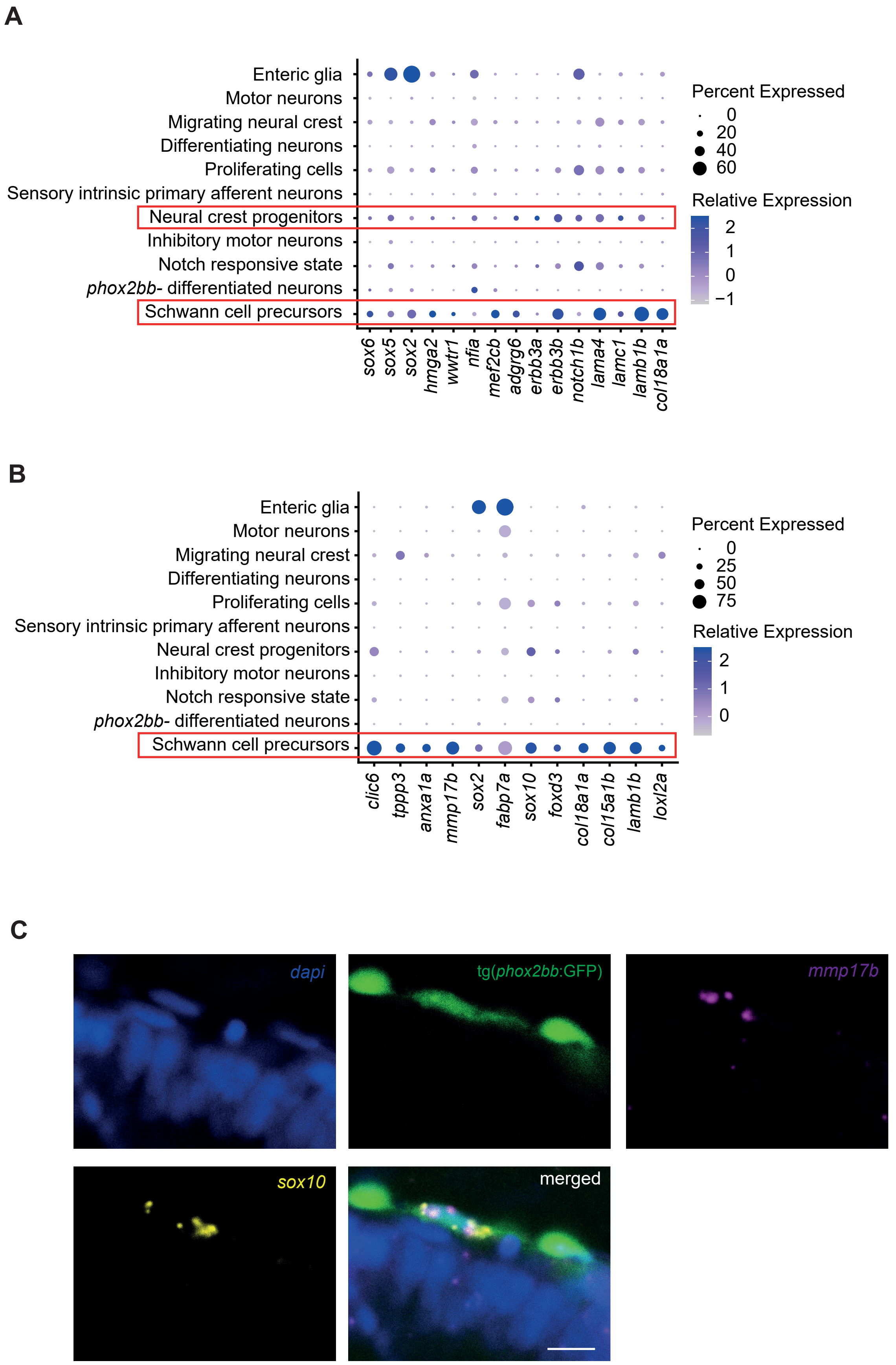

Figure S2. Specific gene expression in progenitor clusters, related to Figure 1
A) Dotplot showing overlap in gene expression in the neural crest precursors cluster and theSCPs cluster.
B) Dotplot showing specific expression of genes in the SCP clusterC) Single plane detailed images of FISH of 5 dpf tg(*phox2bb*:GFP) larvae showing co-localization of dapi (blue), *mmp17b* (magenta), *sox10* (yellow) and *phox2bb* (green) in the intestine. Scale bar represents 4 µm.

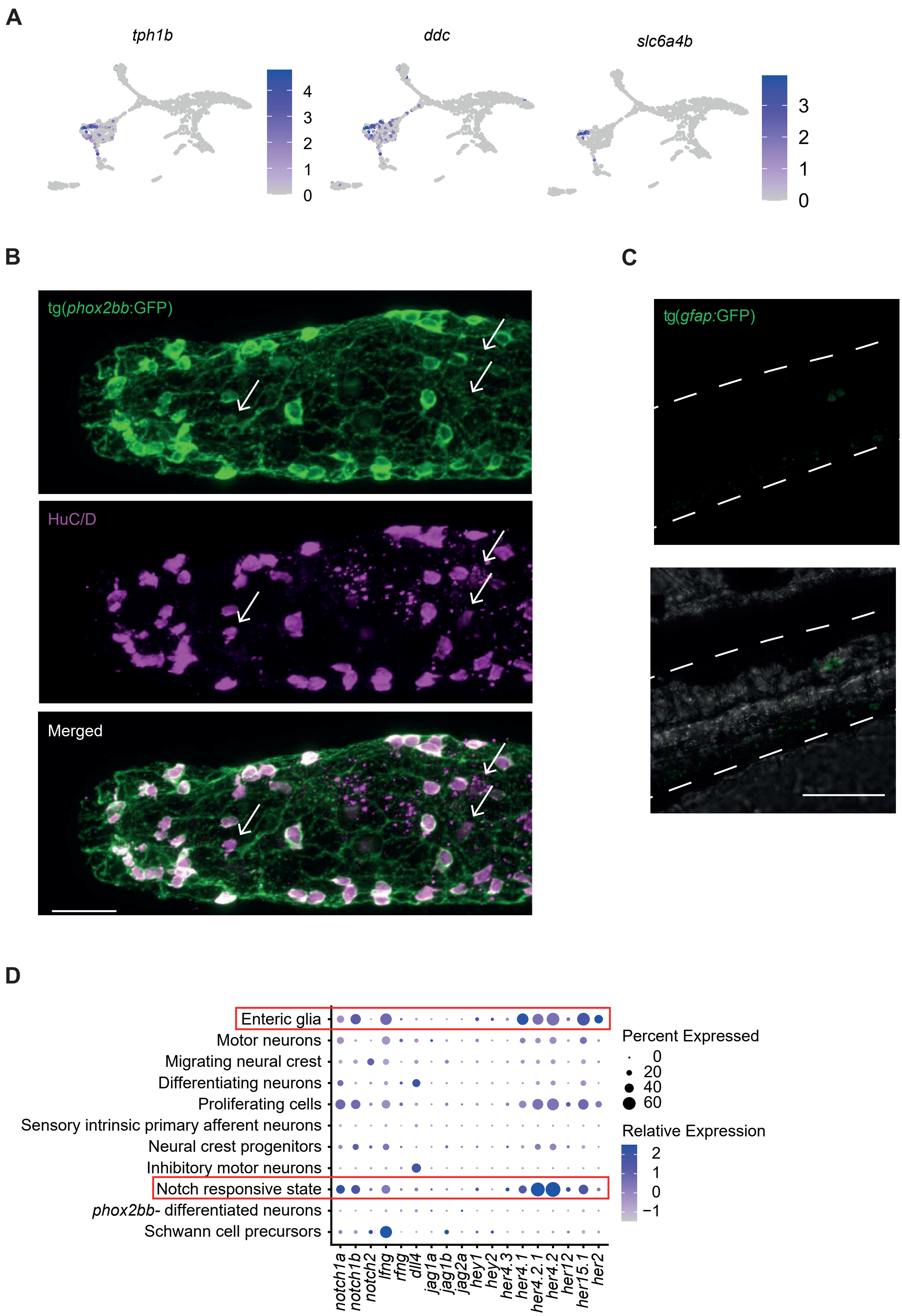

Figure S3. Specific gene expression in enteric glia, notch responsive cells and serotonergic neurons within the inhibitory motor neuron cluster, related to Figure 2

A)Featureplots showing that a subpopulation within the inhibitory motor neuron cluster expresses genes typical for serotonergic neurons. B) Maximum projections of HuC/D and *phox2bb*:GFP double staining on isolated intestines showing that HuC/D+;*phox2bb*- cells (depicted by the arrows) are located in the intestinal tissue. Scale bar represents 50 µm. C) Maximum projection of a live-imaging capture of the tg(*gfap*:GFP) reporter line showing the absence of GFP+ cells in the intestine, which is outlined by dotted lines. Scale bar represents 50 µm. D) Dot plot showing expression of genes in the notch signaling pathway that are specifically expressed in the enteric glia and notch-responsive cells.

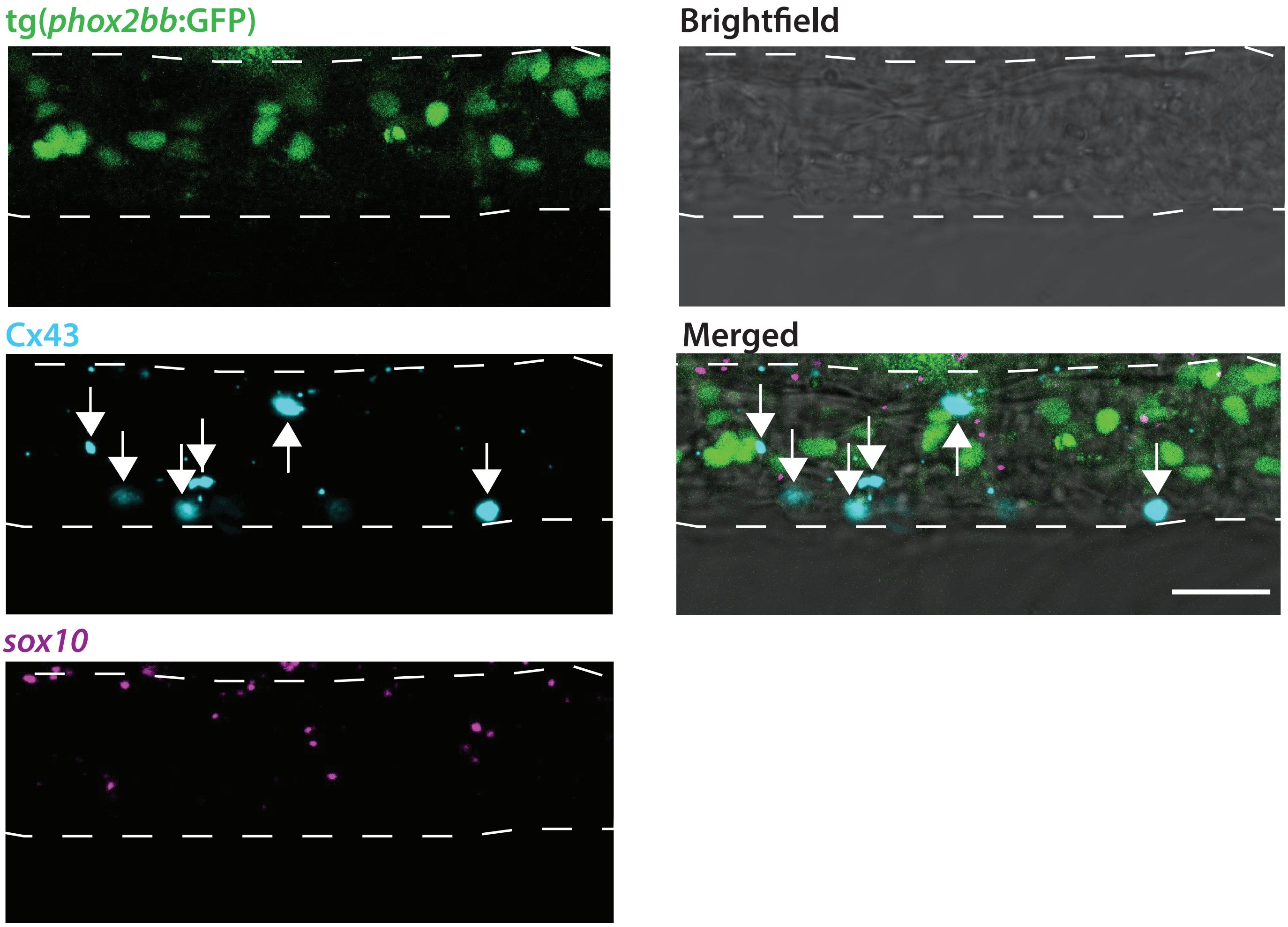

Figure S4. Immunohistochemistry staining of Cx43 in the tg(*phox2bb*:GFP) reporter line in combination with fluorescent in situ hybridization staining of *sox10* shows non overlapping expression in the intestine with Cx43, related to Figure 4.

Representative maximum projections from 5 dpf larvae. Overlap is observed between *sox10* (magenta) and *phox2bb* (green). No overlap is observed between *sox10* (magenta) and Cx43 (cyan), depicted by the arrows. Scale bar represents 23 μm.
